# Rapid molecular assay for the evaluation of clove essential oil antifungal activity against wheat common bunt

**DOI:** 10.3389/fpls.2023.1130793

**Published:** 2023-06-05

**Authors:** Maria Teresa Valente, Laura Orzali, Giuliano Manetti, Francesco Magnanimi, Antonio Matere, Valentino Bergamaschi, Alessandro Grottoli, Sara Bechini, Luca Riccioni, Maria Aragona

**Affiliations:** ^1^ Council for Agricultural Research and Economics (CREA), Research Centre for Plant Protection and Certification (CREA-DC), Rome, Italy; ^2^ Department of Environmental Biology, Sapienza University of Rome, Rome, Italy; ^3^ Department of Biology and Biotechnology “Charles Darwin”, Sapienza University of Rome, Rome, Italy

**Keywords:** *Tilletia laevis*, wheat common bunt, seed borne disease, real time PCR, seed dressing, clove oil, *Triticum durum*

## Abstract

Common bunt of durum wheat (DW), Triticum turgidum L. ssp. durum (Desf.) Husn., is caused by the two closely related fungal species belonging to Tilletia genus (Tilletiales, Exobasidiomycetes, Ustilaginomycotina): Tilletia laevis Kühn (syn. T. foetida (Wallr.) Liro.) and T. caries (DC) Tul. (syn. T. tritici (Bjerk.) G. Winter). This is one of the most devastating diseases in wheat growing areas worldwide, causing considerable yield loss and reduction of wheat grains and flour quality. For these reasons, a fast, specific, sensitive, and cost-effective method for an early diagnosis of common bunt in wheat seedlings is urgent. Several molecular and serological methods were developed for diagnosis of common bunt in wheat seedlings but at late phenological stages (inflorescence) or based on conventional PCR amplification, with low sensitivity. In this study, a TaqMan Real Time PCR-based assay was developed for rapid diagnosis and quantification of T. laevis in young wheat seedlings, before tillering stage. This method, along with phenotypic analysis, was used to study conditions favoring pathogen infection and to evaluate the effectiveness of clove oil-based seed dressing in controlling the disease. The overall results showed that: i) the Real Time PCR assay was able to quantify T. laevis in young wheat seedlings after seed dressing by clove oil in different formulations, greatly reducing times of analysis. It showed high sensitivity, detecting up to 10 fg of pathogen DNA, specificity and robustness, allowing to directly analyze crude plant extracts and representing a useful tool to speed up the tests of genetic breeding for disease resistance; ii) temperature was a critical point for disease development when using wheat seeds contaminated by T. laevis spores; iii) at least one of the clove oil-based formulations tested was able to efficiently control wheat common bunt, suggesting that clove oil dressing could represent a promising tool for managing the disease, especially in sustainable farming.

## 1 Introduction

Common wheat (*Triticum aestivum L*.) and durum wheat (*Triticum turgidum L*.) decay, also known as “wheat bunt”, is a fungal disease that has plagued wheat crops for centuries and is caused by *Tilletia* genus, including different species that both locally and systemically infect wheat crops. There are two main forms of this disease: “common bunt”, caused by *T. laevis* Kühn (synonym *T. foetida*) and *T. caries* Bjerk (synonym *T. tritici*), and “dwarf bunt” of wheat, caused by *T. controversa* Kühn. The distinction between these two diseases was unknown until dwarf wheat caries was identified in 1935 in North America (Young, 1935). To complete the wheat-*Tilletia* scenario it is necessary to consider the partial decay of the grain “karnal bunt” caused by *T. indica* (Mitra, 1931), currently absent in Europe and subject to quarantine regulations. In Italy are mainly present *T. laevis* (80%) and *T. tritici* (9%) which are very similar from both the etiological and morphological point of view: the two species differ only in shape and height of teliospore cell wall ornamentation (Vánky, 2012). However, morphological variants of exospores with intermediate characteristics between *T. laevis* and *T. tritici* have been observed (Flor, 1933; Gassner, 1938; Bremer et al., 1952; Holton, 1954). Those variables probably represent natural hybrids between the two species (Holton, 1944). *Tilletia* species causing common bunt can hybridize, not just with each other, but also with those causing dwarf caries, making taxonomy even more complicated (Holton, 1954; Holton and Kendrick, 1956; Jayawardena et al., 2019). To our knowledge, several DNA-based methods have attempted to distinguish *Tilletia* species, but none have been able to separate *T. caries* and *T. laevis* (Mulholland and Mc Ewan, 2000; Zouhar et al., 2010; Pieczul et al., 2018; Forster et al., 2022). Phylogenetic studies also failed to distinguish these two species and more recently some authors have suggested that *T. caries* and *T. laevis* could be considered as two morphotypes of the same species (Sedaghatjoo et al., 2021; Sedaghatjoo et al., 2022; Forster et al., 2022).

**Figure 1:**
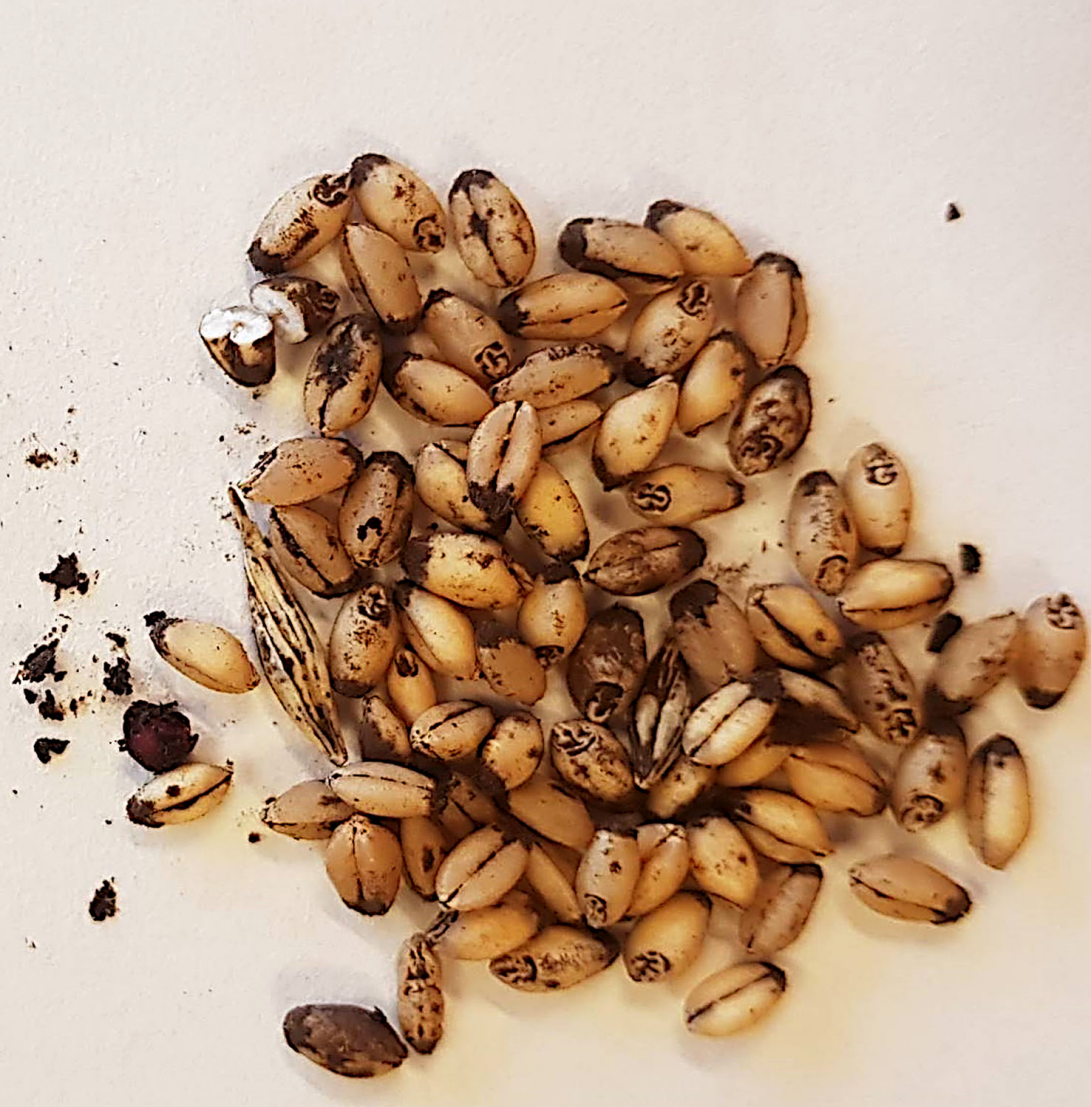
Wheat kernels infected by *T. laevis*. The dark mass of spores is called sorum or false caryopsis.

The fungal infection occurs below the soil surface immediately after seed germination. The teliospores, either in the seed or directly in the soil, germinate, producing infectious hyphae which penetrate the coleoptile. The hyphae initially enter in both resistant and susceptible cultivars, but in the former they do not reach the apical meristem, where they must arrive before the elongation of the internodes so that the disease can develop (Hansen, 1958; Swinburne, 1963). At seed maturity, the caryopsis content is totally composed of a huge mass of spores, called sorum or false caryopsis (**Figure 1**), which breaks down during the harvest, infecting healthy kernels and releasing teliospores onto the soil. Teliospore germination is stimulated by light, and they can remain viable for more than 20 years if stored in a dry atmosphere at room temperature, while, in the soil under natural field conditions, they remain viable for about two years (Woolman and Humphrey, 1924).

Since the second half of 1900’s wheat decay was the cause of substantial losses: the incidence was often higher than 70% and sometimes the whole harvest was destroyed (Cramer, 1967; Jones and Clifford, 1978; El-Naimi et al., 2000). As result of intensive use of chemical seed treatments, together with the use of resistant cultivars, the incidence dropped to less than 1% in Europe. However, the worldwide trading of wheat seeds is causing a new resurgence of this disease, also due to organic farming expansion which promotes the reduction of chemical in favor of biological treatments (Johnsson, 1991; Yarham, 1993) and the return to cultivation of ancient grains, more susceptible to wheat common bunt. It is also important to highlight that the disease incidence is affected by various factors, such as sowing depth, soil moisture, soil temperature, photoperiod, and altitude.

In this scenario, pathogen detection and targeted treatments are of fundamental importance. The easiest way to evaluate the common bunt infection is the sori detection (dark mass of powdery teliospores and host tissue), which are not visible until the ear ripening, occurring late in this host-pathogen interaction. Alternative approaches for direct pathogen detection from infected plants are few and mainly based on the conventional polymerase chain reaction (PCR) techniques (Josefsen and Christiansen, 2002; Eibel et al., 2005). A widely used technique for the disease prevention is seed treatment with authorized products, which have been regulated by phytosanitary product legislation (Reg. CE No. 1107/2009). This technique can be carried out with synthetical chemical products, such as fungicides and/or insecticides that can effectively control the wheat diseases. However, the European Union has been moving towards the replacement of chemicals in favor of substances, such as essential oils (Wilcoxson and Saari, 1996), with a lower environmental impact (Common European Agricultural Policy 2014–2020, CAP, implementing Regulation EU, 2015/408). Essential oils are rich in secondary metabolites, such as alkaloids, phenols, and terpenes, with bio-stimulating activity. These compounds are used by the plants themselves for multiple functions, including abiotic and biotic stress responses, and therefore as a defense mechanism against infections of fungi, bacteria, or insects. The effectiveness of essential oils in plant disease control is well documented (Isman, 2000; Nguefack et al., 2008; Marinelli et al., 2012; Arshad et al., 2014; Sturchio et al., 2014). The potential use in agriculture is further encouraged by their low or no toxicity against vertebrates, easy extractability and eco-compatibility, biodegradability (Zygadlo and Grosso, 1995) and the lack of persistence in soil and water (Misra and Pavlostathis, 1997; Isman, 2000; Isman and Machial, 2006). Indeed, recent studies (Riccioni and Orzali, 2011; Sharma et al., 2017) have shown that essential oils can reduce growth of some pathogenic fungi, and their potential applications in seed treatment are still being studied and developed (Moumni et al., 2021; Kesraoui et al., 2022). To our knowledge, no data are reported in literature on wheat seed treatment with clove essential oil against common bunt under field conditions.

The present work has the following main aims: i) development of a quantitative, early, fast, and low-cost method based on TaqMan Real Time PCR for the highly sensitive detection and quantification of *T. laevis* in young wheat plants, before tillering stage; ii) elucidation of the role of temperature for seed infection success; iii) quantitative evaluation of clove oil-based treatments effectiveness against *T. laevis*, by the molecular method developed. The overall results showed that at least one clove oil-based formulation was able to efficiently control wheat common bunt.

## 2 Materials and methods

### 2.1 Plant and fungal material, treatment compounds and artificial seed inoculation

Pure clove oil stocks and two clove oil-based experimental formulations, named Bioxeda A and Bioxeda B, were provided by Cedax Italia s.r.l. (Forlì, Italy). The two formulations contained micro-encapsulated and nano-encapsulated clove oil, respectively, both at the concentration of 20% (v/v). Two *T. durum* cultivars susceptible to *T. laevis* infection were used: Grifoni 235 and Svevo (gently provided by CREA-CI, Research Centre for Cereal and Industrial Crops). These two cultivars showed the same susceptibility to *T. laevis* infection in preliminary trials carried out by CREA-CI (personal communication). Naturally infected seeds of cultivar Grifoni were used for progressive sowings performed in 2019 and 2020 while, for seed treatments trials, Grifoni and Svevo healthy seeds were artificially inoculated and used in 2020 and in 2022, respectively. *Tilletia laevis* teliospores used for inoculations were collected from sori of Grifoni naturally infected kernels. Their vitality and virulence were evaluated before inoculation. Artificial inoculations for seed dressing tests were performed following Dumalasová and Bartoš (2008) with dry teliospores: briefly, healthy kernels were placed in plastic bags, together with the teliospores, at the dose of 1g of inoculum for 1Kg of seeds resulting in ca 3×10^4^ spores per seed. The envelope was sealed, and the contents manually shaken for 5 minutes allowing the spores to enter in contact with kernel surface, remaining attached to them. The presence of spores was verified by visually checking the kernels under a stereo microscope.

### 2.2 Rapid molecular diagnostic assay

To set-up the early diagnostic assay, based on Real Time PCR, artificially inoculated wheat seedlings were sampled at the stage immediately previous the tillering (from 20 to 40 days from seedling emergence; Feekes growth stage 2.0 – beginning of tillering; Miller, 1992). DNA was extracted from 10 single plants as described by Li et al. (2017). Briefly, 0.5-1 cm of the basal shoot was cut from the seedlings, quickly sterilized in sodium hypochlorite 2% (v/v), rinsed with sterile water and homogenized in 40 µL of lysis buffer (0.5% casein in 10 mM KOH) using a micropestel. Subsequently, the sample was incubated at 95°C for 5 minutes and cooled on ice for 5 minutes, finally, it was centrifuged at 13000 g for 1 minute. The supernatant was recovered into a new tube and the obtained crude extract was used as template for amplification. A TaqMan Real Time PCR assay was carried out using the primers Tri-DL-For (5’-ATTGCCGTACTTCTCTTC-3’), Tri-DL-Rev (5’-GTAGTCTTGTGTTTGGATAATAG-3’) and the dual-labelled probe (5’Cy5-AGAGGTCGGCTCTAATCCCATCA-BHQ2-3’), targeting a specific sequence in the ITS1 region of *T. laevis* and other close related species (*T. caries, T. controversa, T. fusca, T. bromi, T. goloskokovii*). The assay was performed following Tan et al. (2009), modified as described below: the reaction mixture was carried out in 25 μL consisted of 1 × ImmoBuffer (Bioline, London), 5 mM MgCl_2,_ 0.2 mg/mL BSA, 200 mM of each deoxynucleotides, dATP, dTTP, dCTP and dTTP, 1U Immolase DNA polymerase (Bioline) and 200, 400 and 900 nM of each of the dual-labelled probes, the forward and the reverse primers, respectively. The reactions were performed on a CFX96 Real Time PCR system (Bio-Rad Laboratories Inc., CA, USA). The thermal cycling parameters included an initial cycle of 95°C for 10 minutes, followed by 40 cycles of 94°C for 15 seconds, 65°C for 60 seconds with the annealing temperature decreased by 1°C/cycle for 6 cycles to 60°C. To evaluate the presence of PCR inhibitory effects and to verify the amplification efficiency, serial dilutions of purified *T. laevis* DNA, from 10 pg to 1 fg per reaction, spiked with a fixed amount (2 μl) of plant crude extract from *Tilletia*-free wheat plants, were amplified.

### 2.3 Identification and evaluation of the best conditions for *T. laevis* infection

To identify the optimal conditions (photoperiod, temperature, rainfall) for *T. laevis* infection, wheat seeds were artificially inoculated as previously described, and seedlings were grown both outdoor, in pot progressive sowings, and in growth chamber. The term “progressive sowing” refers to a series of repeated sowings deferred at predetermined time points, with the aim of covering the entire period of wheat sowing. For this purpose, five progressive sowings at regular intervals of about two weeks from November to January were carried out for two consecutive years. Each sowing was structured in two pots (pot A and pot B) with 50 inoculated seeds each, located outdoor close to weather station (Vantage Pro2, Davis Instrument, USA) recording temperature, rainfall, and relative humidity.

The time to obtain 50% of seedling emergence (T_50_) was calculated according to Farooq et al. (2005). For each sowing the emerged plants were randomly sampled from both pots. Half of them were used for molecular assay and half for phenotypic evaluation of the presence/absence of the disease symptoms in plants grown to maturity (Feekes growth stage 11.4; Miller, 1992). In the molecular assay a plant was considered infected if produced a positive signal in the Real Time PCR, whereas in the phenotypic evaluation if at least one sorum was present. The disease incidence was calculated by the formula: (Number of infected plants/Total number of analyzed plants) × 100.

From sowing to sampling date, meteorological data (average temperature, minimum temperature, maximum temperature) were recorded every hour; for each day, the average, lowest and highest temperature values were calculated and collected for each day. Days in which the temperature falls within one of these ranges were summed: low-range included temperatures between –5°C and 5°C (range 1), mid-range included temperatures between 5°C and 15°C (range 2) and high-range included temperatures above 15°C (range 3).

To better understand the ideal pedo-climatic conditions that allow fungal infection, Svevo wheat seeds were artificially inoculated as reported above and planted in pots in a growth chamber (KW Apparecchi Scientifici, Siena, Italy). Pedo-climatic conditions were set according to Zscheile jr (1966) and Swinburne (1963): low soil temperatures after sowing (5-10°C), seed vernalization, short photoperiod early in ontogeny, soil humidity between field capacity and drying point and deep sowing at 4-7 cm. Two main sowing conditions were chosen: 1) after seed vernalization; 2) direct sowing, without seed vernalization. For each condition 60 seeds were sowed at 5 cm depth in the soil, in two different pots (30 seeds/pot) kept at 5-10°C up to third-leaf stage. For all the four pots, a 9-hour-light photoperiod was set after seedling emergence. Seed vernalization was carried out before sowing by allowing the seeds to germinate at 27°C for 48 hours (blotter test) and then leaving them at 4°C for 2 weeks. For *T. laevis in planta* detection, 25 seedlings per pot were harvested at the third-leaf stage and analysis was performed as reported in 2.2 paragraph.

### 2.4 Seed dressing

The treatments were applied to inoculated seeds by seed submersion or seed spraying, as detailed in **Table 1**. Seed submersion was carried out by soaking the seeds in the clove oil emulsion (with the addition of 0.05% pinolene as emulsifier) or in the diluted formulations with gentle agitation for 10 minutes; after that they were completely dried on a sterile blotting paper at room temperature in a laminar flow cabinet. Spray treatments were performed with two consecutive applications of the diluted formulations or essential clove oil emulsion with the addition of 0.05% pinolene using a Rotostat (Marline General Engineers Limited, England) seed dressing/coating machine. Pinolene acted as a film-forming compound for improving oil distribution and persistence. Copper sulphate (15.2%) Cutril^®^ (CU) (Serbios s.r.l., Badia Polesine, RO, Italy) was applied by spray in the second season as a further control treatment allowed in organic agriculture (later coded as CU). Sterile water was applied by submersion and used as control to evaluate the washing effect of the seed submersion treatments without active compounds (mechanical effect). The seeds inoculated with *T. laevis* spores as described in 2.1 but not treated (NT), were used as positive control. Not inoculated and not treated seeds were used as negative control. Phytotoxicity of the different treatments was evaluated as the effect on seed germination and seedling emergence by counting the number of established seedlings at the beginning of the secondary tillers arising (Feekes growth stage 2.0; Miller, 1992). The whole experiment was carried out for two growing-crop-productive seasons with cultivar Grifoni in 2020 and Svevo in 2022. For each treatment, three replicated pots, each consisting of 30 and 45 seeds for season 2020 and 2022, respectively, were sowed and placed outdoor with a completely randomized design (CRD). After 14 days from sowing, the number of germinated plants was recorded. Plant sampling was performed at the stage preceding the tillering (from 20 to 40 days from seedling emergence; Feekes growth stage 2.0 – beginning of tillering; Miller, 1992) by cutting plants at the culm base and collecting from each pot at least 5 bulks of 5 plants each; the bulks were then processed for DNA extraction following the protocol described in 2.5 paragraph.

**Table 1:** Treatments of inoculated wheat seeds.

Compound	Commercial product	Application by seed submersion	Application by seed spraying
Copper sulphate	Cutril®	_	110 μl/100 g seed (CU)
Clove oil	Pure essential oil	0.3% for 10 min (CO)	2 consecutive applications at 1% (+ pinolene) (CO_s)
	Bioxeda A	2.5% for 10 min (BioxA)	2 consecutive applications at 5% (+ pinolene) (BioxA_s)
	Bioxeda B	2.5% for 10 min (BioxB)	2 consecutive applications at 5% (+ pinolene) (BioxB_s)
Water		10 min (H_2_O)	_

### 2.5 TaqMan Real Time PCR analysis for *T. laevis* quantification after seed dressing

For seed dressing evaluation, DNA was extracted from plants sampled in the pre-tillering stage (about one month after sowing). From each seedling, a fragment of 4-5 cm was taken from the culm base. Bulks of 5 seedlings each were homogenized in liquid nitrogen; 150-200 mg of powder was lysed in 700 µL of lysis buffer (2% CTAB; 0.2 M Tris HCl pH 7.5; 0.05 M EDTA pH 8; 2 M NaCl) and incubated at 65°C for 10 min. After adding 70 µL of NaAc (3 M, pH 5.2), the samples were kept in ice for 5 minutes and centrifuged at 13.000 g per 10 minutes at 4°C; the supernatant was recovered and treated with RNAse. DNA was purified with 1 volume of chloroform:isoamyl alcohol 24:1 (Sigma-Aldrich, USA) and precipitated with ½ volume of isopropanol. The pellet was resuspended in 200 µL of milliQ water. Finally, DNA was quantified by a Qubit dsDNA HS assay on Qubit 2 fluorometer (Life Technologies™, Invitrogen, USA). A duplex Real Time PCR was set up, using a 18S universal target for fungi and plants (Ioos et al., 2009) as endogenous calibrator to normalize the *T. laevis* qPCR signal to the plant qPCR signal. The reaction was carried out in 20 µL volume, containing SensiMix™ II Probe (Bioline, Taunton, Massachusetts, USA), 0.2 mg/mL BSA, 900 and 400 nM of Tri-DL-For and Tri-DL-Rev primers respectively, 200 nM *T. laevis* probe, 100 nM each 18S primers (18S uni-F, 5’-GCAAGGCTGAAACTTAAAGGAA-3’;18S uni-R, 5’- CCACCACCCATAGAATCAAGA-3’) and 18S probe (5’-JOE-ACGGAAGGGCACCACCAGGAGT-BHQ1-3’) and 10 ng of plant DNA. The thermocycling conditions used were the same applied for the rapid molecular assay. The pathogen biomass was quantified as the ratio of the amplification of *T. laevis* ITS1 relative to the 18S target, which was calculated as 2^−ΔCt^ (Livak and Schmittgen, 2001). To verify the correlation between the number of infected plants and the relative quantification values, 5 bulks with known amounts of infected plants were analyzed.

### 2.6 Data analysis

*Tilletia* infection in progressive sowing assay was evaluated by molecular and phenotypical analysis. The correlation coefficient between the two methods was calculated by Pearson correlation analysis using R software v4.2.2 (R Core Team, 2022) and the package ggpubr v0.5.0 (Kassambara, 2022). A homogeneity test, by a chi-square analysis, was carried out to compare the infection frequencies between the two pots (A and B) both for molecular and phenotypical results.

The meteorological data analysis was performed using a Principal Component Analysis (PCA) biplot, to determine the correlation between temperature and infection. The PCA was performed with R software v4.2.2 (R Core Team, 2022) by using the package factoextra v1.0.7 (Kassambara and Mundt, 2020).

The seed treatments data obtained by Real Time PCR were normalized as described in 2.5 paragraph. Data were then subjected to the analysis of variance (ANOVA) and LSD (least significant difference) test for multiple comparison at *p* = 0.05. To display and compare the results obtained in the two seasons, relative quantification data were balanced to the mean value of the NT control and the proportion to it of the other treatment mean values was calculated: (T/NT) × 100 with T= relative quantification data mean of the treated samples, NT= relative quantification data mean of the not treated samples. The correlation coefficient between the 2020 and 2022 seasons was calculated by Pearson correlation as described above.

For oil phytotoxicity evaluation, the average percentages of established seedlings of 2020 and 2022 seasons were combined after homogeneity evaluation by a chi-square test (data not shown). They were arcsin transformed before ANOVA, followed by Duncan’s test (*p*=0.01), however, mean values were back transformed into percentages for making data reading easier.

## 3 Results

### 3.1 Early molecular diagnosis in progressive sowing test

A Real Time PCR-based method for the early diagnosis of common bunt in wheat seedling has been developed. The qPCR conditions set up in this work showed no inhibition using the crude extract DNA and no differences in the amplification performance between the two extraction methods: the amplification standard curves for both extracts showed an optimal efficiency, a high coefficient of determination (R^2^>0.99) and the same sensitivity, detecting up to 10 fg of target DNA (**Supplementary Figure 1**).

The progressive sowings were evaluated together with infection percentages obtained from molecular and phenotypic assay. The disease incidence values in pot A and pot B were combined after homogeneity evaluation by a chi-square test (**Supplementary Table 1**). In the season 2019/20, the first three sowings (01-03), gave back no bunt infection both in molecular and phenotypic analysis. In the following sowings the infection increased, and the highest values were obtained in the fourth sowing (82% for molecular and 90% for phenotypic analysis), then decreasing by the fifth sowing (22% for molecular and 2.7% for phenotypic analysis). In the season 2020/2021 *T. laevis* infection was recorded for all the sowings; only by phenotypic analysis for sowing n. 07, and only by molecular analysis for sowing n. 10. The highest infection values were found in sowing n. 06 (20% from molecular and 53% from phenotypic analysis) and n. 08 (48% from molecular and 41.4% from phenotypic analysis) (**Table 2**).

**Table 2:** Progressive sowings.

Progressive Sowing(n.)	Sowing date	Seedling emergence		Sampling date for molecular analysis	Infection (%)	
		date	Days after sowing		mol	phen
01	31/10/2019	04/11/2019	4	09/12/2019	0	0
02	14/11/2019	21/11/2019	7	23/12/2019	0	0
03	28/11/2019	08/12/2019	10	20/01/2020	0	0
04	12/12/2019	01/01/2020	20	03/02/2020	82	90
05	03/01/2020	23/01/2020	20	19/02/2020	22	2.7
06	30/11/2020	09/12/2020	9	04/01/2021	20	53
07	14/12/2020	30/12/2020	16	29/01/2021	0	8.6
08	28/12/2020	18/01/2021	21	10/02/2021	48	41.4
09	11/01/2021	29/01/2021	18	01/03/2021	2.7	7.5
10	25/01/2021	05/02/2021	11	18/03/2021	4.9	0

The infection percentage was calculated from Real Time PCR data (mol) and by counting the number of infected plants (phen).

**Figure 2:**
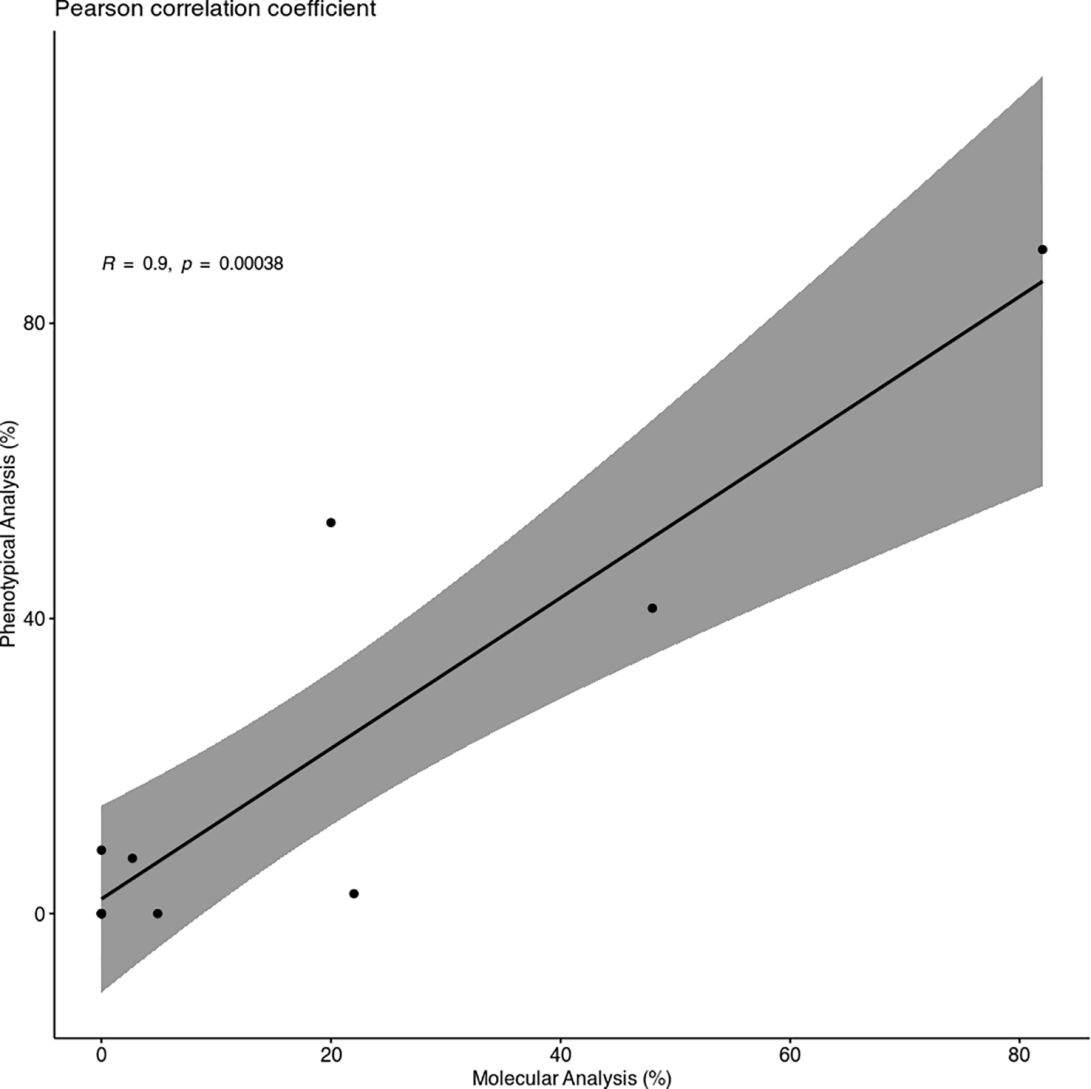
Scatter Plot of the Pearson correlation coefficient. The plot shows a strong and positive correlation between the infection percentages of Molecular (MA) and Phenotypical Analysis (PA) (R = 0.9) and a *p* < 0.01.

Despite some minor differences between the two different analyses, the Pearson correlation coefficient showed a strong correlation between them, with a coefficient R of 0.9 (**Figure 2**).

### 3.2 Role of temperature in infection success

With the aim of understanding the optimal *in vivo* conditions for *T. laevis* infection, the meteorological conditions during the growth period from sowing to the second true leaf stage (about 30 days) were analyzed: this period represents the limit phase within which the seedlings are considered susceptible to infection to *Tilletia* spp. (Gaudet et al., 2007).

To assess and discriminate if specific temperature ranges play a role in the infection process, data were analyzed by using the PCA biplot (Data in **Supplementary Table 2**). This method geometrically analyzes information in multivariate data. When the variable positions are close, there is a high correlation, moreover, the acute angle between each pair of variables indicates a positive correlation, while the obtuse angle indicates a negative correlation (Jolliffe and Cadima, 2016).

**Figure 3:**
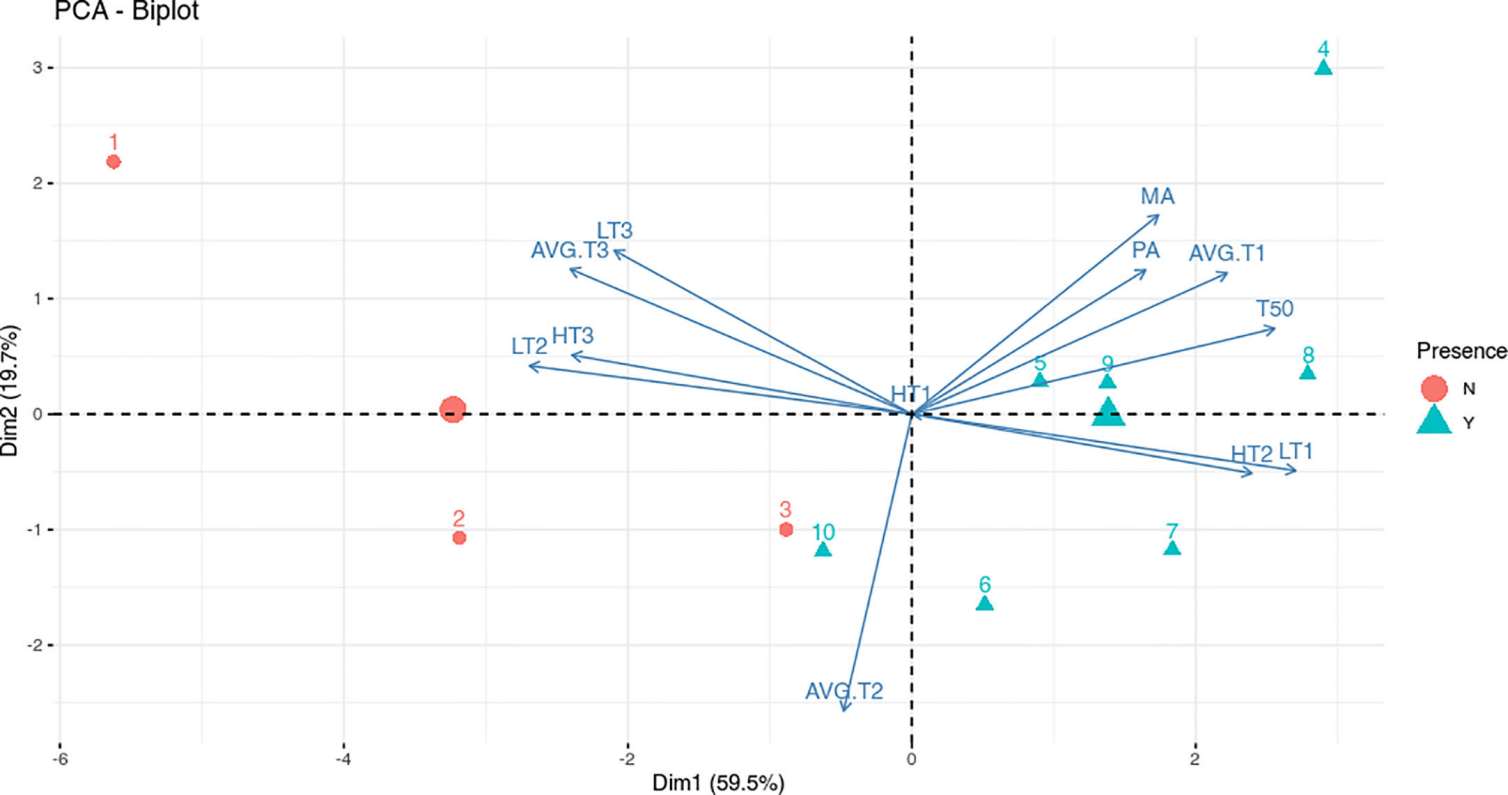
Biplot for the principal component analysis (PCA) of meteorological data and infection analyses. Red dots indicate sowings that did not show disease (N) while blue triangles indicate sowings that showed disease (Y). The numbers indicate sowings while the red dot and the blue triangle with no number indicate the group centroid. The arrows indicate the contribution of variables to the two axes of the PCA: number of days at Highest Temperature (HT), Lowest Temperature (LT) and Average Temperature (AVG.T); Molecular Analysis (MA) and Phenotypical Analysis (PA) percentages and median germination time (T_50_). The temperature ranges considered are indicated on arrows variables with 1, 2 or 3 (1: -5/5°C; 2: 5/15°C; 3:15/25°C).

The disease incidence data, from both molecular and phenotypical analyses (MA and PA), T50 and all the variables indicating the number of the days with lower temperature (AVG.T1, LT1 and TH2) were well negatively correlated with the variables indicating the number of the days with higher temperature (AVG.T3, LT2 and HT3) (**Figure 3**). The negative correlations between the number of the days with higher and lower temperature, together with the distribution of the sowings point on the PC1, suggest a significant role of the temperature in the infection process. As shown in **Figure 3**, the sowings dates were well separated on the PCA axis 1 (59.5% of variance) in two groups: presence or absence of infection. Moreover, the greater the days with low temperatures, the higher the molecular and phenotypical infection rates. In addition, T_50_ had a positive correlation with higher number of days at lower temperatures and higher infection rates. The percentages reported on the axis referred to the variability explained by the principal component. There was no correlation between all variables and number of days with an average temperature ranging from 5 to 15°C (AVG.T2); no day was recorded with the maximum temperature below 5°C (TH1) (**Figure 3**).

### 3.3 Growth chamber assay

Wheat is a winter cereal and *T. laevis* needs low temperatures to infect this crop, so we tested the *in vivo* fungal infection capability in the typical winter temperature range of 5-10°C. The main goal of the experiment performed in growth chamber was to achieve a high level of seedling infection also in controlled conditions, in order to be able to replicate the method for seed dressing and sowing tests. In both seed vernalization and direct sowing, *T. laevis* infection rates were comparable (approx. 70%), suggesting that seed vernalization did not affect the early infection. The infection rates obtained were in agreement with those reported in literature (Wilcoxson and Saari, 1996).

### 3.4 Relative quantification of *T. laevis* by real time PCR

**Figure 4:**
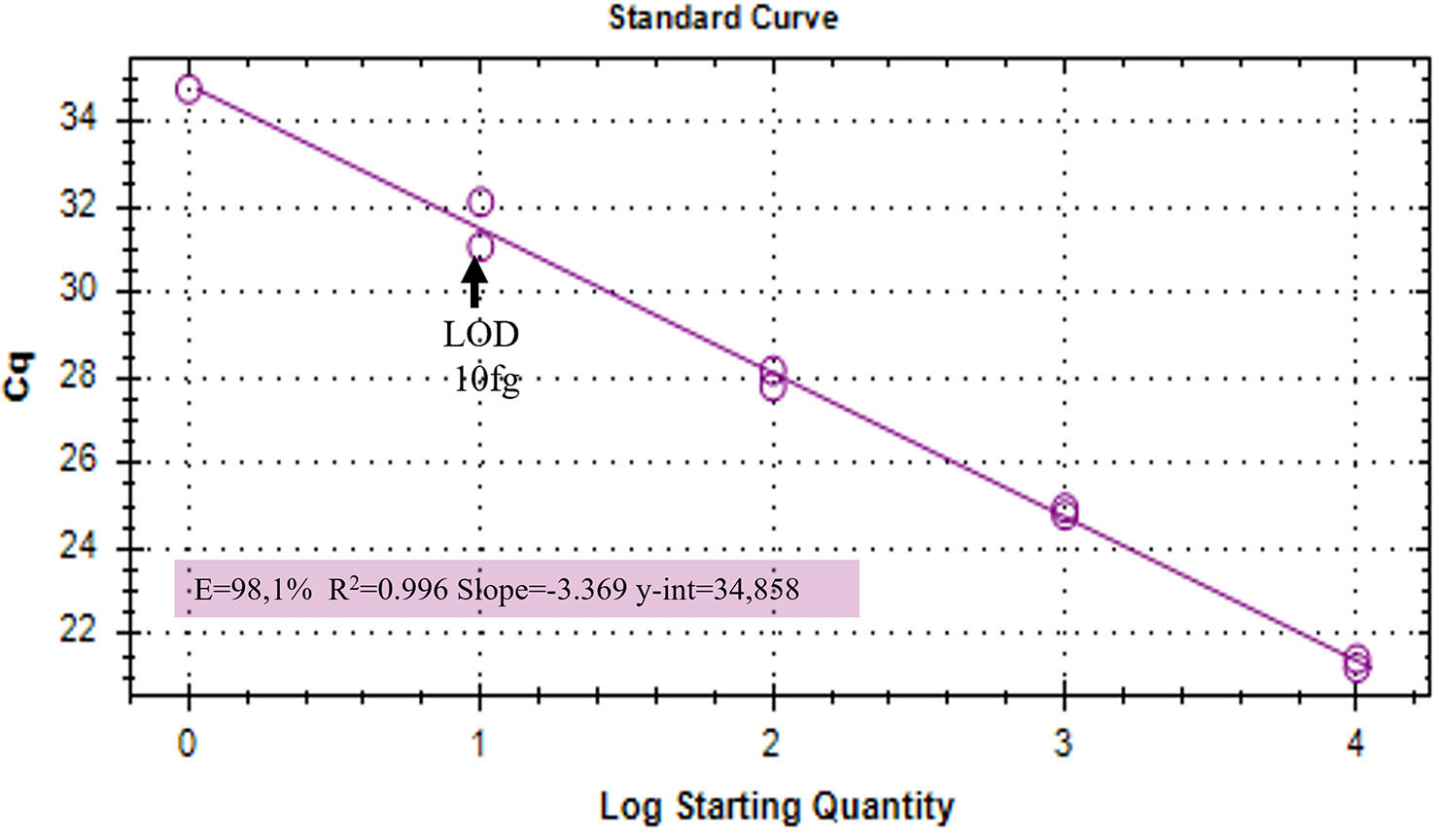
Amplifications of serial dilutions of *T. laevis* DNA starting from 10000 fg. LOD, Limit Of Detection.

**Figure 5:**
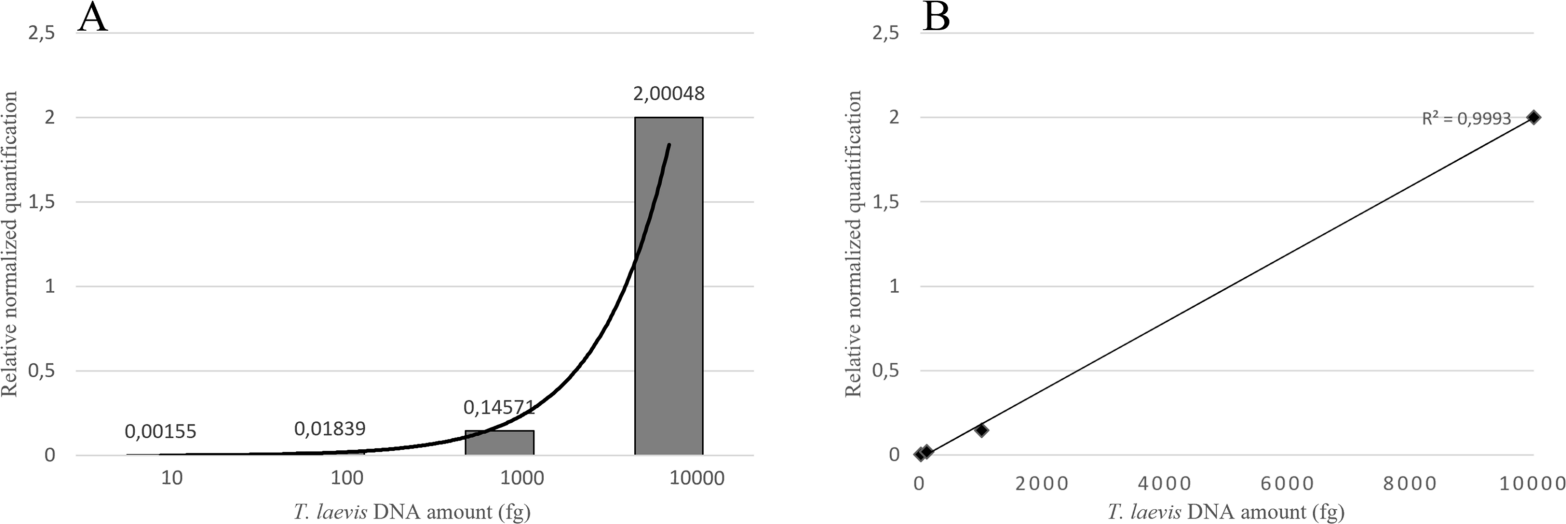
Target normalized quantification of serial dilution of *T. laevis* DNA **(A)** and best fit correlation between the relative quantification values and the known amounts of target DNA **(B)**.

Duplex qPCR showed both very good efficiency and coefficient of determination (R^2^>0.99), and a limit of detection (LOD) of 10 fg of *T. laevis* DNA per reaction (**Figure 4**). Relative quantification, showing the amount of target amplicon (*T. laevis*) normalized to the total DNA (18S), correlated efficiently with the amounts of target DNA. Normalized amplifications corresponded to an exponentially increasing trend of about an order of magnitude (**Figure 5A**), and the best fit curve, correlating the data of normalized expression signal to the known amounts of *T. laevis* DNA, showed a good linear correlation (R^2^ = 0.99) (**Figure 5B**). Furthermore, the presence of the 18S endogenous calibrator guaranteed greater reliability in the quantification, representing an internal amplification control, and allowing the precise quantification of total DNA used as template in the reaction.

**Figure 6:**
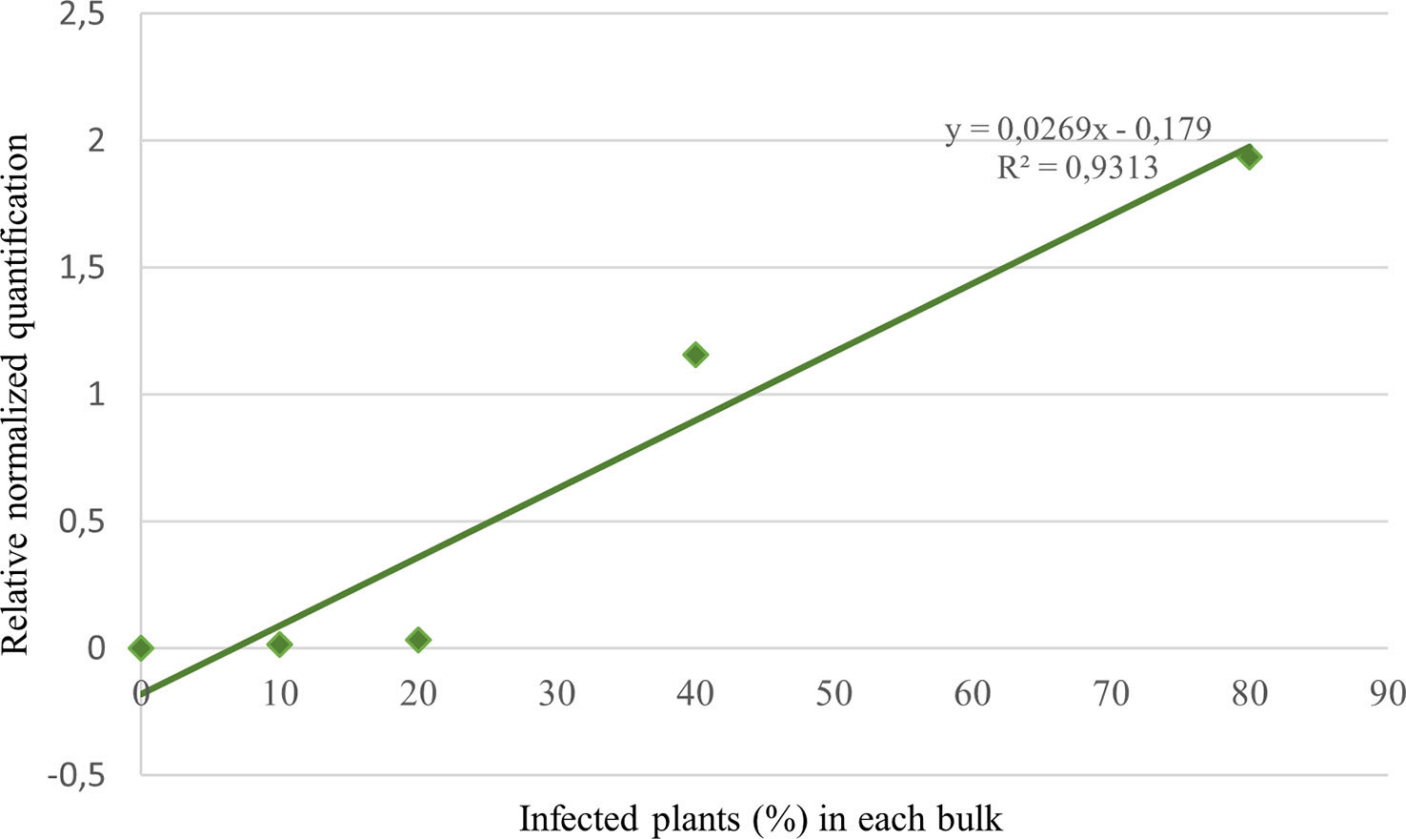
Target normalized quantification of 5 bulks consisting in 10 wheat seedlings each with a known number of infected plants (0, 1, 2, 4, 8).

The correlation between relative quantification and disease incidence was verified by analyzing bulks with a known number of infected plants (R^2 ^= 0.93; **Figure 6**). To compare the infection success between treatment assays in seasons 2020 and 2022, the equation resulting from the interpolation line of the aforesaid data was used to obtain an approximate percentage of infected plants in the untreated control tests. In 2020 the average infection of NT plants resulted to be about 50%, versus 20% in 2022.

### 3.5 *Tilletia laevi*s quantification and phytotoxicity evaluation after seed dressing

**Figure 7:**
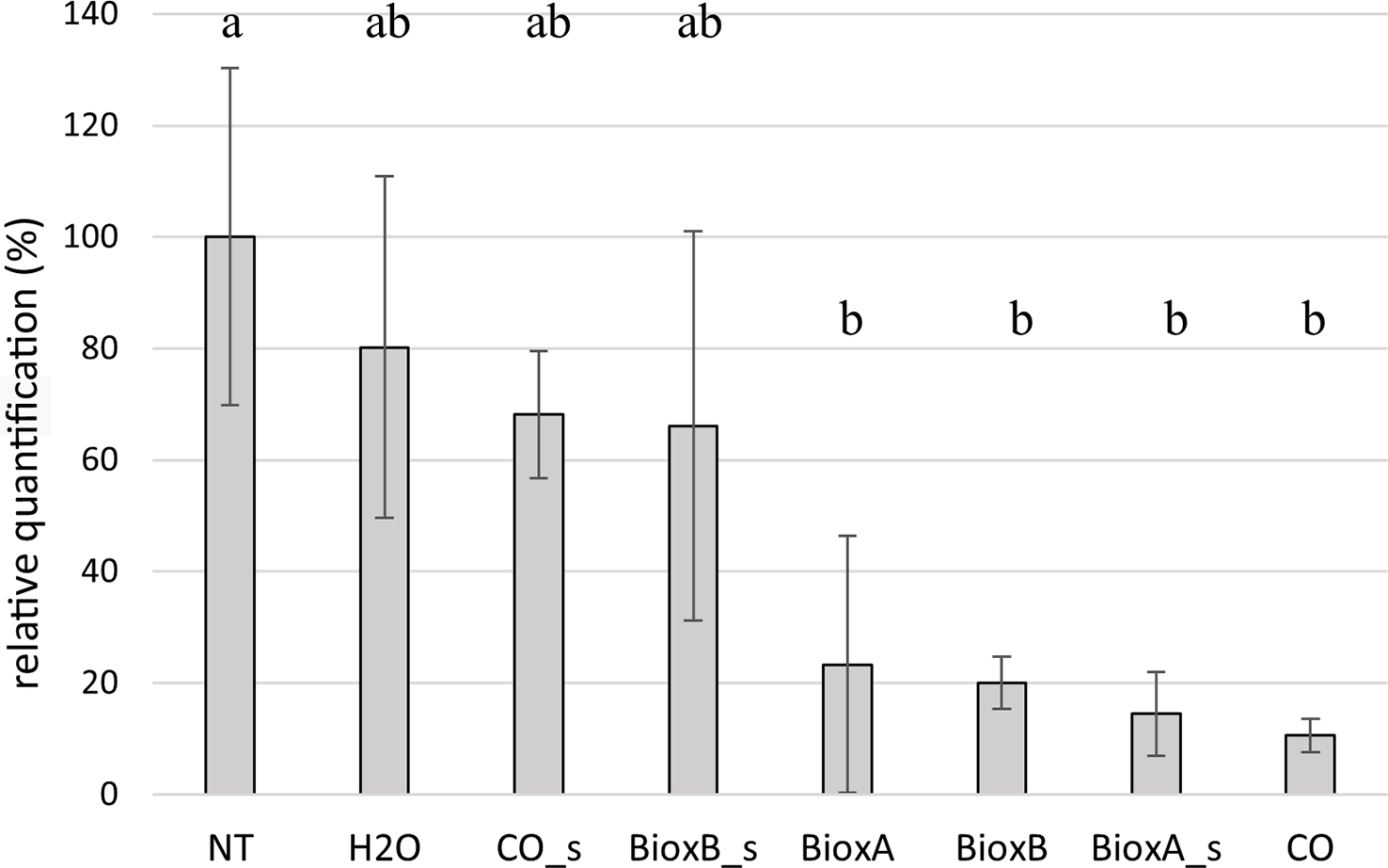
Barplot of season 2020. Relative quantification, expressed as percentage referring to NT as 100%, is reported on the y-axe. The values obtained from the different treatments are the mean of three replications (± standard error). Significant differences (p = 0.01) found using the LDS test for multiple comparison are indicated as letters a–b; the same letter means no statistical significance. NT, Not treated; H_2_O, water; CO, clove oil; BioxB, Bioxeda B; BioxA, Bioxeda A; s, spray treatment.

**Figure 8:**
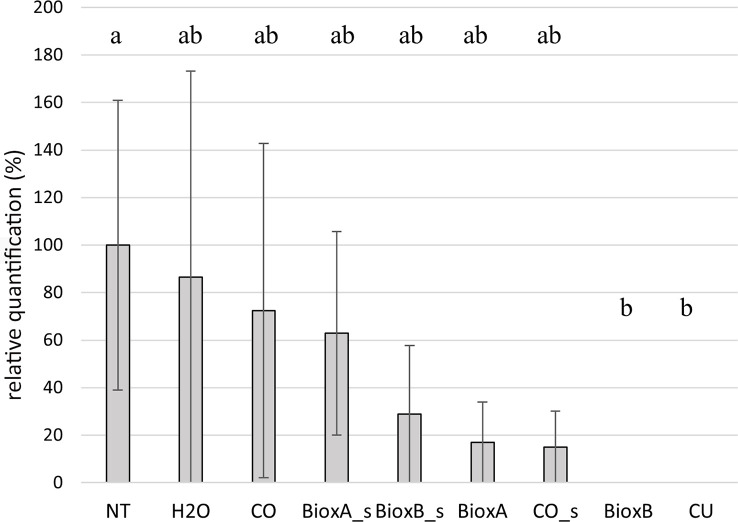
Barplot of season 2022. Relative quantification, expressed as percentage referring to NT as 100%, is reported on the y-axe. The values obtained from the different treatments are the mean of three replications (± standard error). Significant differences (p = 0.01) found using the LSD test for multiple comparison are indicated as letters a–b; the same letter means no statistical significance. NT, Not treated; H_2_O, water; CO, clove oil; BioxB, Bioxeda B; BioxA, Bioxeda A; s, spray treatment; CU, Cutril^®^.

*Tilletia laevis* quantification in seedlings deriving from dressed seeds was performed by TaqMan Real Time PCR. Data were analyzed considering the two growing seasons separately (**Figures 7**, **8**). The seed treatments were generally able to control bunt infection. In season 2020, significant differences were observed between the treatments performed by submersion (BioxA, BioxB and CO) and NT control. Among the spray treatments, only Bioxeda A gave results significatively different from the NT control, while the other two (Clove oil spray and Bioxeda B spray), together with water treatment, were comparable to NT control. Water treatment has produced a reduction of infection too, probably due to the water washing effect on the teliospores present on the kernel surface (**Figure 7**). Negative control (not treated and not inoculated) always gave negative results (data not shown).

In season 2022, the Bioxeda B submersion treatment showed the best results as no *T. laevis* infection was recorded, as well as with the chemical treatment (CU) used as control. The other treatments showed a lower infection rate compared to NT control, suggesting a protective (or just washing) effect against the pathogen, however no significant differences were found (**Figure 8**). In this season, expression data of the whole experiment, including the NT control, were lower than in season 2020, and no correlation was found between the average infection rates of the tested oil treatments of 2020 and 2022 seasons (R^2^ = 0.09; **Supplementary Figure 2**).

The phytotoxicity of the different treatments used for seed dressing was evaluated as effect on seed germination and seedling emergence, assessing the number of established seedlings before the secondary tillers arising. Data collected from the two seasons were merged and analyzed together: significant differences were found only for clove oil treatment by immersion (CO), compared to NT control (**Table 3**). This treatment showed a toxic effect on wheat seeds significantly reducing germinability and seedling emergence. A significant correlation was found from the analysis of 2020 and 2022 phytotoxicity data (R^2^ = 0.94; **Supplementary Figure 3**), indicating the reproducibility in the different seasons.

**Table 3:** In pot seedling emergence at the beginning of the secondary tillers arising (Feekes growth stage 2.0) for treatments phytotoxicity evaluation.

Emerged plants (%)				
Treatments	2020	2022	Mean of the two seasons	Duncan’s
BioxA	85.7 ± 5.7	89.3 ± 2.3	87.5 ± 2.5	a
BioxB	87.6 ± 4.3	89.3 ± 1.2	88.5 ± 1.2	a
CO	13.3 ± 5.9	11.3 ± 6.1	12.3 ± 1.4	b
H20	78.1 ± 12.9	88.0 ± 2.0	83.0 ± 7.0	a
BioxA_s	88.6 ± 11.4	88.0 ± 4.0	88.3 ± 0.4	a
BioxB_s	76.2 ± 9.2	84.0 ± 5.3	80.1 ± 5.5	a
CO_s	84.8 ± 6.6	74.0 ± 20.8	79.4 ± 7.6	a
NT	82.9 ± 4.9	81.3 ± 2.0	82.1 ± 1.1	a

Values are reported as mean of three replications for each season ± standard deviation. Treatments associated with different letters differ according to Duncan’s multiple range test at p ≤ 0.01. NT, Not treated; H2O, water; CO, clove oil; BioxB, Bioxeda B; BioxA, Bioxeda A; s, spray treatment.

## 4 Discussion

The TaqMan Real Time PCR represents the assay of choice in diagnosis of plant diseases, compared to conventional PCR and, in this work, it was confirmed its high degree of sensitivity, specificity and reproducibility. The development of a molecular method based on Real Time PCR for the early diagnosis of common bunt in wheat seedling, which to date and to our knowledge is not available, was an important goal of this work. The excellent sensitivity, detecting up to 10 fg of pathogen DNA, the specificity and robustness of the assay, thanks to which it was possible to directly analyze the crude plant extract without lowering the performance levels, guaranteed the effectiveness of the method. Furthermore, the possibility of working on a crude extract, without the need to purify the DNA with the use of commercial kits or more expensive protocols in terms of time and costs, guaranteed an easy, fast and cheap application. The comparable results obtained from molecular and phenotypic analyses showed the efficacy of this method and it could represent a useful tool especially in breeding tests for genetic resistance or in seed treatments evaluation trials, where the waiting periods for results are rather long: 8-9 months for phenotypic symptom analysis compared to 1-2 months required for molecular analysis. Previous works have developed molecular and serological methods for diagnosis of common bunt in wheat seedlings but at later phenological stages, starting from the inflorescence, or based on conventional PCR amplification, with a very low sensitivity (Josefsen and Christiansen, 2002; Eibel et al., 2005). Real Time PCR allows to precisely quantify fungal biomass and therefore can be used in the assessment of plant infection, evaluating the degree of susceptibility/tolerance (Wessling and Panstruga, 2012; Anderson and McDowell, 2015; He et al., 2020).

In this study, the developed duplex Real Time PCR assay, combined with an optimized DNA extraction protocol, has proved to be an effective tool for accurate quantification of the target pathogen in plant material and it was successfully used for evaluating the effectiveness of clove oil-based seed dressing in controlling the disease. The method was able to quantify *T. laevis* in young wheat seedlings after seed dressing with clove oil in different formulations and ways of application, significantly reducing the time of analysis. Correlation between the amount of *T. laevis* DNA and the percentage of infected plants, with relative quantification, showed the accuracy of the method.

To set up the most favorable conditions for wheat seed infection by *T. laevis*, we evaluated the influence of temperature for the success of infection process, for subsequently testing the efficacy of seed dressing by different clove oil-based formulations. In literature, different works reported a positive correlation between the frequency of common bunt infected spikes caused by seedborne *Tilletia* teliospores and low temperatures. For example, Johnsson (1992) reported that the common bunt infection of winter wheat in field experiments was correlated with climate data, but only for the critical period of 1-11 days after sowing. The infection was most severe when the average temperature during this period was 6-7°C. In alignment with Johnsson (1992) and Koprivica et al. (2009), in this work the critical role of temperature as daily average, maximum and minimum temperatures, in the infection process has been confirmed in the progressive sowing assay. PCA analysis showed that both molecular and phenotypical infection were well correlated with the number of days with an average temperature between -5 and 5°C, while there was a negative correlation with the number of days with an average temperature between 15 and 25°C. These correlations confirmed the important role of low temperature in *T. laevis* infection process. The sowings were well separated into two groups: presence or absence of infection, so the low temperature lasting for more than 7-10 days could be related to the disease, but probably other factors, such as wheat cultivar susceptibility and external factors could repress or enhance the infection. Another interesting point concerns the influence of temperature during the germination period for fungal tissue infection. Koprivica et al. (2009) showed that the lower the temperature, the longer the wheat germination time, giving the pathogen more time to reach the apical meristem tissue. In this work, the clove oil was tested on durum wheat as seed treatment against *T. laevis* during two seasons, by using different compositions (pure oil and as main component of two Bioxeda experimental formulations) and two different types of seed dressings (submersion and spray). Since no correlation was found between the 2020 and 2022 treatments (average infection frequency of the untreated plants in 2022 experiments was lower than in 2020), the seed treatment data were analyzed and discussed separately for the two seasons. This low degree of infection did not allow the evaluation of treatments effect compared to season 2022 where the differences among NT and treatments were more pronounced. In the season 2020 all the submersion treatments, regardless of the product/formulation used, showed an evident positive effect significantly reducing *Tilletia* plant infection, as well as the spray treatment with Bioxeda A. In season 2022, the best results were performed by the Bioxeda B submersion treatment, as no *T. laevis* infection was recorded.

In previous works, essential clove oil has been shown to be effective against various fungal and bacterial pathogens *in vitro* (Rabadia et al., 2011; Duduk et al., 2015; Orzali et al., 2020), but few data have been published on its efficacy *in vivo* and even fewer under field conditions. An *in vivo* laboratory study showed that *Fusarium* spp. infection on wheat seeds was reduced after soaking the kernels in clove essential oil solutions at various doses (Grzanka et al., 2021). Rice seeds immersion treatment with clove essential oil was found to reduce by 50% the disease incidence caused by the bacterium *Burkholderia glumae* (Sari et al., 2022). Field pea seed treatments (spray and submersion) with clove oil-based formulations were tested for efficacy against artificially inoculated Ascochyta blight fungi under field conditions, although variability between years was found (Riccioni et al., 2019). The clove oil, applied as soil treatment in *F. oxysporum* f. sp. *lycopersici* infected soil, showed a very strong inhibitory effect causing significant reduction of wilt disease in tomato plants (Sharma et al., 2017). Clove oil was also tested as a post-harvest treatment: maize seeds fumigation with clove oil for at least 24 h was found to protect the seeds against *Aspergillus flavus* during storage (Boukaew et al., 2017); green mold (*Penicillium digitatum*) growth and symptomatology in navel oranges was controlled by clove oil applied on post-harvested fruits as microemulsions or in vapor phase (He et al., 2016). To our knowledge, no data are available in literature on wheat seed treatment with clove essential oil against common bunt under field conditions.

In conventional agriculture, wheat common bunt disease can be well controlled by chemical fungicide seed treatment, but in sustainable farming these fungicides are no longer allowed, and reduction of synthetic pesticides, whenever possible, has been mandatory in EU since 2014 (Romanazzi et al., 2022). To date, there are two clove oil-based products with fungicidal activity, already available in the market and allowed for post-harvest fruit treatments, but none is registered for wheat common bunt control (http://www.fitosanitari.salute.gov.it/fitosanitariws_new/FitosanitariServlet). Previous studies on alternative and eco-friendly control methods of common bunt have mainly focused on different seed treatments, e.g. with plant extracts from *Cannabis sativa*, *Eucalyptus globulus*, *Thuja sinensis* and *Datura stramonium*, cereal flour, milk powder and other organic compounds, hot water, hot air treatments and antagonistic bacteria and fungi, achieving good results in controlling the disease (Singh et al., 1979; Borgen et al., 1995; El-Naimi et al., 2000; Shams-Allah et al., 2016). Oil application on seeds deserves deep attention, as different treatments may give different results. When considering submersion treatment, it is important to take in account also the eventual phytotoxicity, which depends not only on the oil, but also on the dose, the timing of application and the crop (Somda et al., 2007; Orzali et al., 2014). Since no information about clove oil phytotoxicity on durum wheat seeds is available, the 0.3% clove oil dose tested in this work, was chosen from data obtained from other crops (Riccioni et al., 2016; Orzali et al., 2020). For example, it was reported that for tomato seeds the 0.3% clove oil submersion treatment for 10 minutes did not affect germination while the 0.4% concentration had negative effects, even if in that case the germination reduction was quite exiguous, around 10% (Orzali et al., 2020). The fumigation treatment of maize seeds with 0.5% clove essential oil had negative effects on seed germination, stem length and root length (Boukaew et al., 2017). In the current research, the 0.3% clove oil concentration was found to be toxic for durum wheat seeds by submersion application, as the number of emerged plants was significantly lower than the untreated plants, with more than 80% reduction in germination. On the other hand, spray treatments at the same concentration did not show any phytotoxic effects.

In presence of *T. laevis* seed inoculum, our data showed that seed treatment is therefore recommended to control this pathogen. Use of clove essential oil (by submersion and spray application) showed an interesting potential in the control of wheat common bunt disease, albeit with fluctuating efficacies related to environmental conditions and pathogen inoculum pressure. In fact, it turned out that it is difficult to predict the outcome of infection, which is linked to climatic conditions. These results were very promising because, if antifungal efficacy of clove oil dressing will be further confirmed, it could represent a useful, effective, natural, and ecological alternative to chemical pesticides in wheat common bunt control. Concerning the two application methods, the spray treatment requires lower formulation volumes, allowing the use of lower amounts of active ingredients in favor of an economically sustainable industrial process. On the other hand, specific devices and equipment are required and a lower reproducibility was observed in this kind of application. The submersion process demonstrated a more reliable effectiveness over the years, but it is anyway considered cumbersome and time-consuming when treating large amounts of seeds on a large scale, due to a larger volume of liquid and subsequent drying (Afzal et al., 2020). Phytotoxicity is also an important aspect to consider when treating seed by dipping at concentrations greater than 0.3%. Both submersion and spray methods can be considered as valid tools for the application of clove oil (preferably in formulated form) and the choice of application may depend on the advantages and disadvantages that should be evaluated at industrial/farm level.

In conclusion, the developed diagnostic method proved to be a fast, specific, sensitive, and cost-effective tool for early diagnosis of common bunt in wheat seedling. Thanks to these features, it could also be useful in early screening for resistance, taking advantage of the possibility of assessing the occurrence of infection even at early stages without waiting for the symptoms of the ear to fully ripen stages. This is the first report on the effectiveness of wheat seed dressing by natural oil-based treatments for controlling common bunt disease. Further studies are needed with experimental trials in open field to compare and validate the results obtained in this first pot trial-based research. Finally, in future works the search of other natural oils for controlling *T. laevis* with efficacy and low phytotoxicity, is highly desirable and necessary.

## Data availability statement

The original contributions presented in the study are included in the article/**Supplementary Material**. Further inquiries can be directed to the corresponding author.

## Author contributions

Conceptualization, LR and MA. Molecular analysis, MV, GM, FM, VB and SB. Statistical analysis, AG, GM and LO. Infection tests and treatments assay, LO, FM, VB, GM and AM. Writing - original draft preparation, MA, MV, LO and AG with contributions from GM, FM, SB, VB and AM. Writing - review and editing, MA, MV, LO and AG. All authors contributed to the article and approved the submitted version.

## In memoriam

This paper is dedicated to the memory of Luca Riccioni, who conceived and started this work with dedication and passion.

## Supplementary material

Supplementary Figure 1 Amplification performance of rapid molecular assay.

Supplementary Figure 2 Scatter Plot of Pearson correlation coefficient between the *T. laevis* relative quantification of tested oil treatments in 2020 and 2022 seasons.

Supplementary Figure 3 Scatter Plot of Pearson correlation coefficient between phytotoxicity frequencies for each treatment in 2020 and 2022 seasons.

Supplementary Table 1 Chi-square analysis comparing the infection ratios between pot A and pot B for each sowing and diagnostic assay.

Supplementary Table 2 molecular and phenotypical analysis, meteorological and germination mean time data used for statistical analysis.
