# Supplementary figures and images for: Rapid molecular assay for the evaluation of clove essential oil antifungal activity against wheat common bunt
Source: Front Plant Sci. 2023 Jun 5;14:1130793. doi: 10.3389/fpls.2023.1130793 (PMC10277744; doi:10.3389/fpls.2023.1130793)

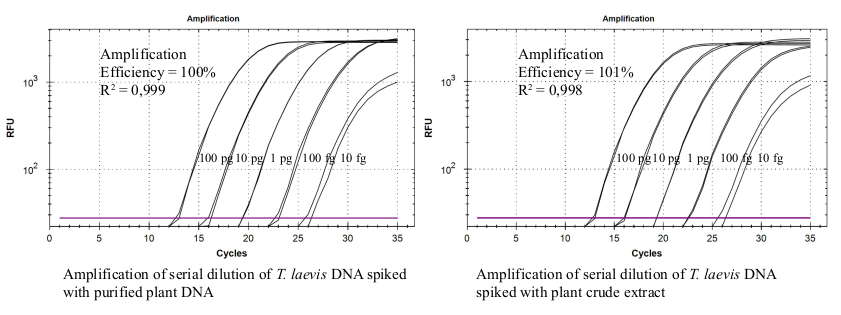

Supplement: Supplementary Figure 1 — Amplification performance of rapid molecular assay. [file Image_1.tif]

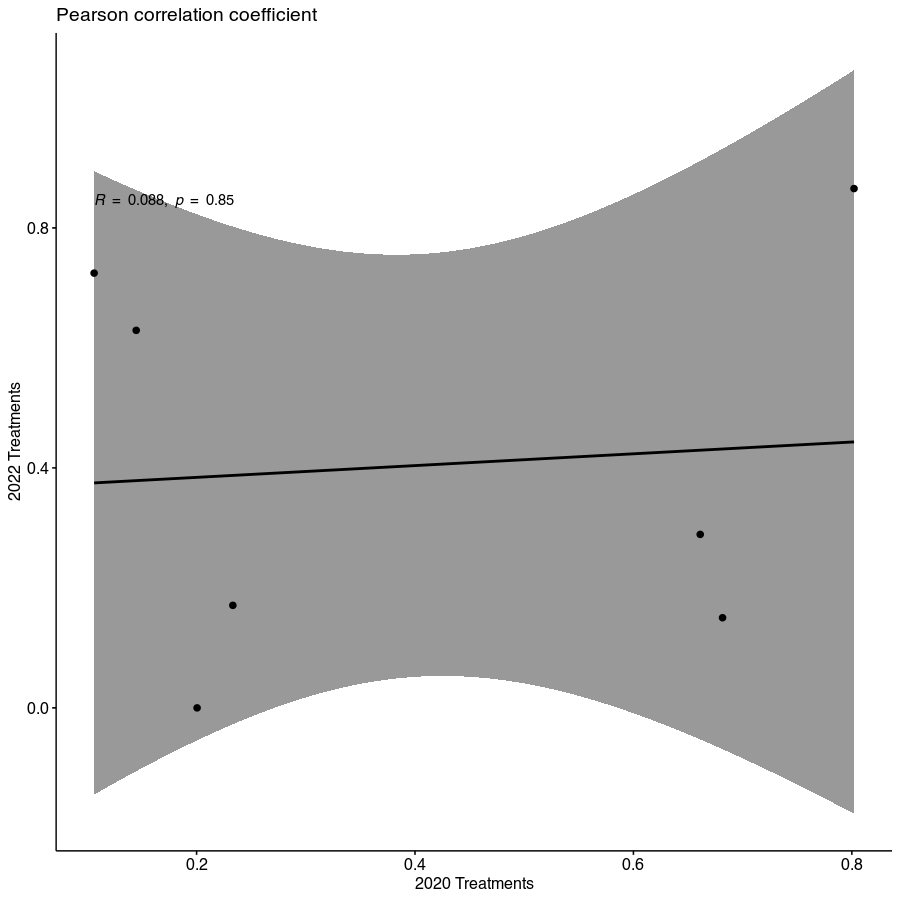

Supplement: Supplementary Figure 2 — Scatter Plot of Pearson correlation coefficient between the T. laevis relative quantification of tested oil treatments in 2020 and 2022 seasons. [file Image_2.tif]

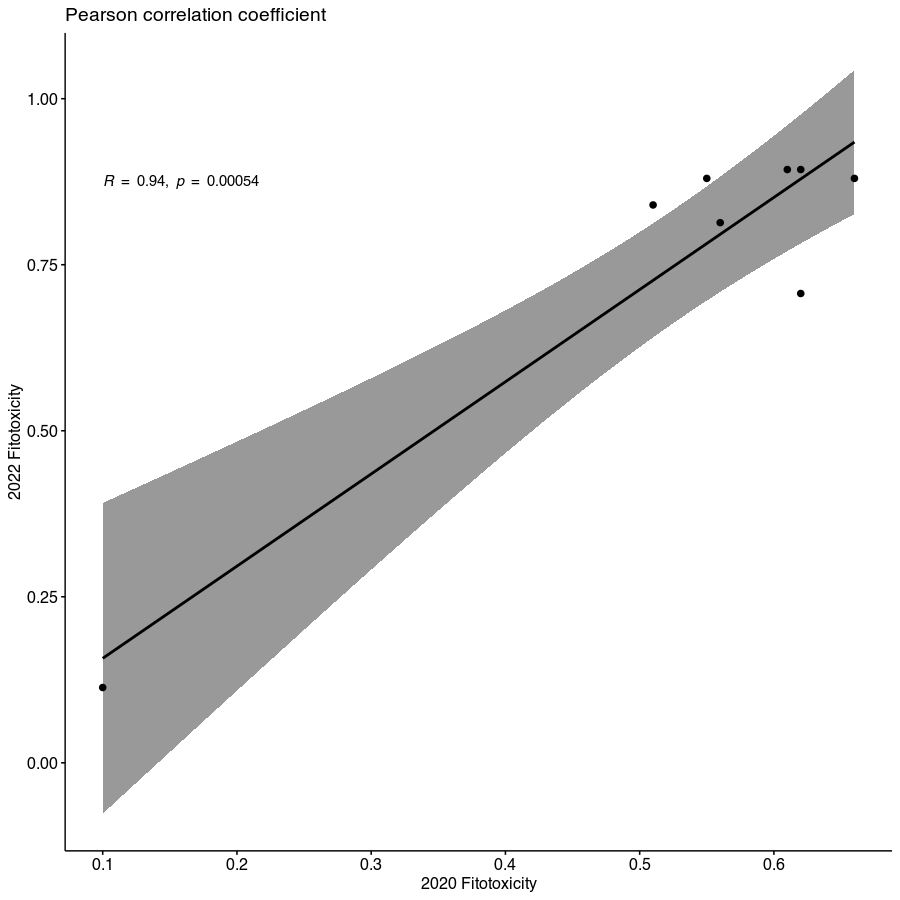

Supplement: Supplementary Figure 3 — Scatter Plot of Pearson correlation coefficient between phytotoxicity frequencies for each treatment in 2020 and 2022 seasons. [file Image_3.tif]
