# Supplementary material for: Rapid molecular assay for the evaluation of clove essential oil antifungal activity against wheat common bunt
Source: Front Plant Sci. 2023 Jun 5;14:1130793. doi: 10.3389/fpls.2023.1130793 (PMC10277744; doi:10.3389/fpls.2023.1130793)
Supplement: Supplementary Table 1 — Chi-square analysis comparing the infection ratios between pot A and pot B for each sowing and diagnostic assay. [file DataSheet_1.pdf]

Sowing date	Molecular assay			Phenotypic assay		
	Infected plants/analysed plants		p-value	Infected plants/analysed plants		p-value
	Pot A	Pob B		Pot A	Pob B	
31/10/2019	0/25	0/25	/	0/25	0/25	/
14/11/2019	0/25	0/25	/	0/25	0/25	/
28/11/2019	0/25	0/25	/	0/25	0/25	/
12/12/2019	20/25	21/25	0,71	13/15	15/16	0,51
03/01/2020	5/25	6/25	0,73	1/18	0/19	0,30
30/11/2020	6/25	4/25	0,48	12/24	14/25	0,67
14/12/2020	0/25	0/25	/	2/22	2/25	0,89
28/12/2020	11/25	13/25	0,57	6/15	6/14	0,88
11/01/2021	1/19	0/19	0,31	2/20	1/20	0,55
25/01/2021	1/20	1/21	0,97	0/16	0/14	/
