# Supplementary material for: Rapid molecular assay for the evaluation of clove essential oil antifungal activity against wheat common bunt
Source: Front Plant Sci. 2023 Jun 5;14:1130793. doi: 10.3389/fpls.2023.1130793 (PMC10277744; doi:10.3389/fpls.2023.1130793)
Supplement: Supplementary Table 2 — molecular and phenotypical analysis, meteorological and germination mean time data used for statistical analysis. [file DataSheet_2.pdf]

Supplementary_table 2

Sowing	AVG-T1	AVG-T2	AVG-T3	LT1	LT2	LT3	HT1	HT2	HT3	MA	PA	Presence	T50
S01	0	30	10	2	36	2	0	5	35	0	0	N	4
S02	0	37	3	8	32	0	0	8	32	0	0	N	7
S03	4	35	1	22	18	0	0	13	27	0	0	N	10
S04	11	28	1	31	9	0	0	17	23	82	90	Y	20
S05	8	32	0	27	13	0	0	14	26	22	2.7	Y	20
S06	0	39	1	28	12	0	0	22	18	20	53	Y	9
S07	6	34	0	33	7	0	0	26	14	0	8.6	Y	16
S08	7	33	0	28	12	0	0	29	11	48	41.4	Y	21
S09	10	30	0	25	15	0	0	22	18	2.7	7.5	Y	18
S10	4	36	0	25	15	0	0	11	29	4.9	0	Y	11

AVG T1	Days with an average temperature between -5 and 5 °C
AVG T2	Days with an average temperature between 5 and 15 °C
AVG T3	Days with an average temerature between 15 and 25 °C
LT1	Days with a lower temperature between -5 and 5 °C
LT2	Days with a lower temperature between 5 and 15 °C
LT3	Days with a lower temperature between 15 and 25 °C
HT1	Days with a higher temperature between -5 and 5 °C
HT2	Days with a higher temperature between 5 and 15 °C
HT3	Days with a higher temperature between 15 and 25 °C
MA	Molecular analysis percentages
PA	Phenotypic analysis percentages
Presence	Presence of infection in MA or PA (Y: presence; N: absence)
T50	Median germination time
